# Bidirectional synaptic mechanisms of ocular dominance plasticity in visual cortex

**DOI:** 10.1098/rstb.2008.0198

**Published:** 2008-10-31

**Authors:** Gordon B. Smith, Arnold J. Heynen, Mark F. Bear

**Affiliations:** Howard Hughes Medical Institute, The Picower Institute for Learning and Memory, Department of Brain and Cognitive Sciences, Massachusetts Institute of Technology Cambridge, MA 02139, USA

**Keywords:** ocular dominance plasticity, long-term potentiation, long-term depression, BCM theory, metaplasticity

## Abstract

As in other mammals with binocular vision, monocular lid suture in mice induces bidirectional plasticity: rapid weakening of responses evoked through the deprived eye followed by delayed strengthening of responses through the open eye. It has been proposed that these bidirectional changes occur through three distinct processes: first, deprived-eye responses rapidly weaken through homosynaptic long-term depression (LTD); second, as the period of deprivation progresses, the modification threshold determining the boundary between synaptic depression and synaptic potentiation becomes lower, favouring potentiation; and third, facilitated by the decreased modification threshold, open-eye responses are strengthened via homosynaptic long-term potentiation (LTP). Of these processes, deprived-eye depression has received the greatest attention, and although several alternative hypotheses are also supported by current research, evidence suggests that α-amino-3- hydroxy-5-methyl-4-isoxazolepropionate (AMPA) receptor endocytosis through LTD is a key mechanism. The change in modification threshold appears to occur partly through changes in *N*-methyl-d-aspartate (NMDA) receptor subunit composition, with decreases in the ratio of NR2A to NR2B facilitating potentiation. Although limited research has directly addressed the question of open-eye potentiation, several studies suggest that LTP could account for observed changes *in vivo*. This review will discuss evidence supporting this three-stage model, along with outstanding issues in the field.

## 1. Introduction

The substrate for binocular vision in mammals is the convergence of retinotopically matched inputs onto common postsynaptic cortical neurons. Development, refinement and maintenance of these binocular connections depend on the quality of visual experience. Degrading vision in one eye—a manipulation called monocular deprivation (MD)—shifts the ocular dominance (OD) of cortical neurons such that they cease to respond to stimulation of the deprived eye. This phenomenon of OD plasticity occurs during early post-natal development in all mammals with binocular vision, and in some species (e.g. the mouse) it persists well into adulthood. In humans, lasting visual impairment can result from several conditions that degrade or unbalance vision prior to adolescence, including strabismus, uncorrected refractive errors and cataracts (Doshi & Rodriguez 2007).

The significance of understanding the synaptic and molecular bases of OD plasticity cannot be overstated. First, the processes revealed by OD plasticity are likely to be the same as those that refine cortical circuitry in response to the qualities of sensory experience during development, and thus determine the capabilities and limitations on visual performance in adults. Second, rapid OD plasticity is an example of cortical receptive field plasticity, the most common cellular correlate of memory in the brain. It is therefore likely that understanding the mechanisms of OD plasticity will yield insight into the molecular basis of learning and memory. Third, the detailed understanding of how synaptic connections are weakened by sensory deprivation will suggest possible strategies to reverse such changes, and possibly overcome amblyopia. Finally, the detailed understanding of how synaptic connections are strengthened by experience will suggest possible strategies to augment such changes, and promote recovery of function after brain injury.

Mouse visual cortex has emerged as the favoured preparation for the mechanistic dissection of OD plasticity. First, mice display robust OD plasticity in response to MD, and the kinetics and behavioural consequences of OD plasticity are very similar to those observed in others species. Second, the property of binocularity is established early in cortical processing by convergence of thalamic inputs onto layer 4 neurons, potentially simplifying the analysis of the underlying synaptic changes. Third, mice are genetically homogeneous and plentiful, enabling rapid progress using coordinated biochemical and electrophysiological studies *in vitro* and *in vivo*. Fourth, the absence of a columnar organization makes feasible the use of chronic recordings from awake animals. Fifth, the fact that the mouse visual cortex is relatively undifferentiated (e.g. compared with monkey V1) suggests that insights gained here might apply broadly across species and cortical areas. Sixth, genes can be delivered or deleted in the mouse visual cortex by genetic engineering or viral infection. Finally, mice have emerged as valuable models of human genetic disorders, offering the opportunity to use the powerful paradigm of OD plasticity to understand how experience-dependent cortical development can go awry in genetic disorders and, hopefully, suggest ways that these disorders could be corrected.

## 2. Bidirectional kinetics of OD plasticity in the mouse

Responsiveness of the mouse visual cortex to stimulation of the eye has been measured using a number of methods, including single-unit recordings, visually evoked potentials (VEPs), optical imaging of intrinsic signals related to oxygen usage, immediate early gene expression and imaging of cellular calcium transients. Each of these methods introduces a different bias. For example, recording of spiking activity is biased towards deep layers of cortex, where units are the easiest to isolate from one another; VEP recordings reflect summed synaptic currents that are dominated by thalamocortical input to layer 4; and calcium imaging is limited to neurons within a few 100 μm of the cortical surface. These differences are important to recognize, as it has become clear that the mechanisms of OD plasticity vary according to layer, as we will discuss. However, despite this caveat, all methods yield a consistent picture of what happens when one eyelid is closed.

Visual responses of mice raised in a normal (laboratory) visual environment are dominated by the contralateral eye. Even in the binocular segment, the contralateral eye response is approximately double the ipsilateral eye response. This OD is rapidly shifted when the contralateral eyelid is closed at approximately four weeks of age. A shift of the contra/ipsi ratio is detectable with as little as 1 day of MD and reaches an asymptote by 3 days, when the contra/ipsi ratio is approximately 1 (Frenkel & Bear 2004; Liu *et al*. 2008).

The VEP recording method has been particularly useful for understanding the processes that account for this shift. A practical advantage of the VEP is that it can be recorded with chronically implanted electrodes. Thus, before- and after-MD measurements can be made from the same mice, and the data can be collected from awake alert animals. Applying this VEP technique to varying periods of MD in mice demonstrated that the observed OD shift towards the non-deprived eye results from the combination of two distinct processes: a weakening of deprived-eye responses and a strengthening of open-eye responses (Frenkel & Bear 2004). Although both processes contribute to the overall OD shift, they do so with distinct time courses (Frenkel & Bear 2004). Three days of MD produces a strong shift in the contra/ipsi ratio by weakening the response of the deprived (contralateral) eye without affecting the non-deprived (ipsilateral) eye responses. By contrast, 7 days of deprivation yields both deprived-eye depression and potentiation of open-eye responses. This pattern of rapid deprived-eye depression and delayed open-eye potentiation has also been noted in other species (Mioche & Singer 1989).

Importantly, the bidirectional consequences of MD are each functionally significant. In rats, the deprived eye exhibits a dramatic reduction in visual acuity assessed through visually guided behaviour following MD (Prusky *et al*. 2000; Iny *et al*. 2006). In the same visually guided task (Iny *et al*. 2006), open-eye performance was enhanced following MD, indicating that the bidirectional plasticity of VEPs reflects functionally meaningful changes in sensory processing.

Based on the synthesis of theoretical and experimental work, a comprehensive model of OD plasticity has been proposed (Frenkel & Bear 2004): (i) deprived-eye responses depress via homosynaptic long-term depression (LTD), (ii) the plasticity threshold, determining the boundary between potentiating and depressing input activity, becomes lower in response to the decreased cortical activity that follows monocular lid closure, and (iii) open-eye responses potentiate via homosynaptic long-term potentiation (LTP) due to the lower threshold for synaptic potentiation. In the sections that follow, we will briefly present the data that support this model. Alternative hypotheses for deprived-eye depression and open-eye potentiation will be discussed later in the review.

## 3. LTD as a molecular mechanism of deprived-eye depression in the mouse

MD triggers response depression in cortex by degrading images on the retina, not by eliminating retinal activity (Rittenhouse *et al*. 2006). The adequate stimulus for response depression appears to be the weakly correlated afferent activity arising in the visually deprived retina, and relayed to cortex by the lateral geniculate nucleus (Bear 2003; Blais *et al*. 2008). Synaptic modifications that are driven by activity in the affected inputs are said to be ‘homosynaptic’. Thus, there has been a search for mechanisms of homosynaptic LTD of excitatory synaptic transmission. This search has been aided by the development of LTD paradigms that use electrical stimulation of synaptic transmission in brain slices or *in vivo*. An important caveat is that it is abundantly clear that the mechanisms of LTD vary from one synapse type to the next, so one must be cautious in generalizing (Malenka & Bear 2004).

The ‘canonical’ LTD mechanism in the CA1 region of hippocampus was used to guide early studies in the visual cortex. In CA1, weak activation of NMDA receptors (NMDARs) activates a postsynaptic protein phosphatase cascade that alters the phosphorylation state of AMPA receptors (AMPARs), which are in turn internalized by clathrin-dependent endocytosis (Malenka & Bear 2004). These changes can be detected biochemically using phosphorylation site-specific antibodies and assays of receptor surface expression. The biochemical signature of LTD can be used as a ‘molecular fingerprint’ to ask whether similar changes occur in visual cortex following a period of MD. To date, this has only been examined in the rat visual cortex, but the results support the hypothesis that MD induces this type of LTD in visual cortex (Heynen *et al*. 2003).

**Figure 1:**
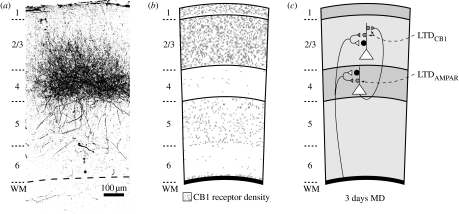
Mechanisms of deprived-eye depression vary across cortical layers. (*a*) Thalamocortical axons project to both layers 4 and 3 of the mouse visual cortex. Axons were labelled with biotin-conjugated dextran (3000 MW) injected into binocular dLGN of a P28 mouse. Imaging took place 4 days later from fixed coronal sections containing visual cortex. Laminar borders were determined based on Nissl staining. Image courtesy of J. Coleman. (*b*) Schematic showing CB1 receptor density variations across cortical layers, with high levels of expression in supragranular layers and limited expression in layer 4. Drawing based on Deshmukh *et al*. (2007). (*c*) Three days of MD produces depression of deprived-eye responses in both layers 3 and 4 through distinct mechanisms. In layer 3, both LTD and deprived-eye depression require CB1 activation, whereas LTD is independent of postsynaptic AMPAR internalization. The absence of high CB1 expression in layer 4 correlates with the lack of a requirement for CB1 activation in both LTD and deprived-eye depression. By contrast, LTD in layer 4 requires AMPAR endocytosis, suggesting that deprived-eye depression at this synapse may also occur through AMPAR internalization.

A second approach to address whether LTD is induced by MD is to ask whether naturally occurring synaptic depression *in vivo* occludes LTD *ex vivo*. As originally pointed out by Nigel Daw and colleagues, however, the mechanisms of LTD appear to vary according to cortical layer (Daw *et al*. 2004). This issue has been recently examined in the mouse by Crozier *et al*. (2007). Identical stimulation protocols applied to the radial inputs to layers 4 and 3 induced LTD via activation of postsynaptic NMDARs. However, only in layer 4 was the LTD mediated by clathrin-dependent AMPAR endocytosis. Conversely, only in layer 3 was the LTD sensitive to pharmacological blockade of cannabinoid receptors. However, in both layers, the LTD measured in slices was reduced (occluded) by 3 days of MD *in vivo*. Thus, the evidence suggests that MD induces LTD in both layers 3 and 4, but by distinct molecular mechanisms (figure 1).

The evidence is very strong that MD leads to LTD of synaptic transmission in visual cortex. However, still controversial is the question of the relative contribution of this change to the functional consequences of MD (i.e. cortical blindness). An approach to this question has been to correlate deficits in LTD and OD plasticity in genetically or pharmacologically modified mice. However, limitations inherent to this approach are that the manipulations may affect only the stimulation requirements for LTD in brain slices, not the core mechanism; compensatory adaptations may occur; and plasticity may be disrupted *in vivo* by alterations in retina, thalamus or behavioural state. Furthermore, such studies have not taken into account key features of visual cortical plasticity: *first*, that an OD shift can occur by deprived-eye depression, open-eye potentiation or both (Sawtell *et al*. 2003; Frenkel & Bear 2004), and *second*, that the mechanisms of LTD (Crozier *et al*. 2007) and OD plasticity (Liu *et al*. 2008) vary according to layer (figure 1).

The OD shift assayed using single-unit recordings (layers not specified) is disrupted in the glutamic acid decarboxylase 65 (GAD65) knockout mouse, which has impaired cortical inhibition (Hensch *et al*. 1998*a*). Although drifting baseline recordings obscured the deficit in the original report (Hensch *et al*. 1998*a*), layer 3 LTD is also clearly impaired in these mice (Choi *et al*. 2002). Similarly, cannabinoid receptor blockade prevents both LTD (Crozier *et al*. 2007) and deprived-eye response depression in unit recordings restricted to layer 3 (Liu *et al*. 2008).

On the other hand, a dissociation of LTD and OD plasticity was suggested in several protein kinase A mutants. For example, the RIβ knockout mouse reportedly has a deficit in layer 3 LTD but exhibits a normal OD shift after 4 days of MD (Hensch *et al*. 1998*b*). Unfortunately, the significance of the LTD deficit is unclear as control recordings in WT mice were not performed under these experimental conditions. Two additional studies deleting either of the RII subunits of PKA further complicate the relationship between PKA, LTD and OD plasticity. RIIα KO mice display normal LTD in layer 3, whereas both LTP in this preparation and OD plasticity were moderately reduced (Rao *et al*. 2004). By contrast, RIIβ KO mice exhibit normal LTP at the same synapse, but lack both LTD and OD plasticity (Fischer *et al*. 2004).

Given that many different plasticity mechanisms exist in the visual cortex (Daw *et al*. 2004), it is likely that a large portion of these seemingly conflicting results may be attributable to laminar differences between the molecular pathways supporting LTD and LTP. In mice, MD produces an OD shift simultaneously in layers 4 and 3 (Liu *et al*. 2008), suggesting that the disruption of layer-specific plasticity mechanisms (Wang & Daw 2003; Rao & Daw 2004) will affect OD plasticity in a complex fashion. Many studies using single-unit recordings pool neurons recorded across all layers, thereby preventing analysis of layer-specific deficits in plasticity. In addition, the use of acute single-unit recordings in many studies precludes the separation of mechanisms impacting the loss of deprived-eye responses from those affecting potentiation of the open eye, because eye-specific responses cannot be compared before and after deprivation. In KO mice with abnormalities in both LTP and LTD this can be especially problematic, as it becomes impossible to determine the process that contributes to the observed OD phenotype.

If we restrict consideration to layer 4, where VEP recordings are made, and to periods of MD 3 days or less, when the shift is dominated by deprived-eye depression, the data support the hypothesis that MD shifts OD via the loss of AMPARs at visually deprived synapses. However, it remains to be determined whether this is the only—or the most important—mechanism for deprived-eye response depression.

## 4. Metaplasticity during MD

After approximately 5 days of contralateral eye MD, the ipsilateral (non-deprived) eye responses begin to grow. Because there has been no change in the quality of visual experience through this eye, there must be an adaptation in the cortex that allows response potentiation. A theoretical framework for this aspect of OD plasticity was provided by the influential Bienenstock, Cooper and Munro (BCM) theory (Bienenstock *et al*. 1982). According to this theory, the reduction in overall cortical activity caused by closing the contralateral eyelid decreases the value of the modification threshold, *θ*_m_, thereby facilitating potentiation of correlated inputs (reviewed by Bear 2003).

In accordance with theoretical predictions, experiments using a period of dark rearing to decrease activity in the visual cortex have demonstrated that the threshold level of stimulation required to induce LTD and LTP is modifiable by prior visual experience. In both rats and mice, 2 days or more of darkness is sufficient to shift *θ*_m_, moving the boundary between LTP and LTD induction towards lower stimulation frequencies (Kirkwood *et al*. 1996; Philpot *et al*. 2003, 2007). Brief re-exposure to light rapidly reverses the effects of dark rearing on the modification threshold (Kirkwood *et al*. 1996; Philpot *et al*. 2003).

Modifications of NMDAR function were proposed as a physiological mechanism for changing *θ*_m_ (Bear *et al*. 1987; Abraham & Bear 1996), and a number of recent studies have focused specifically on the ratio of NR2A to NR2B subunits (Bear 2003). Rats that are dark reared or exposed to the dark for brief periods show reductions in the ratio of NR2A to NR2B proteins, which can be reversed rapidly upon re-exposure to light (Quinlan *et al*. 1999*a*,*b*). Additionally, dark rearing increases the decay times of synaptic NMDA currents (Carmignoto & Vicini 1992), while also increasing the sensitivity to NR2B selective antagonists and temporal summation of synaptic responses (Philpot *et al*. 2001).

These findings are consistent with an increased proportion of NR2B-containing NMDARs at synapses, and demonstrate that the changes observed at the protein level have a meaningful effect on synaptic transmission. The longer decay kinetics of NR2B-containing receptors have been proposed to facilitate the summation of inputs and thereby promote coincidence detection, possibly facilitating LTP (Monyer *et al*. 1994; Flint *et al*. 1997). In addition, NR2B subunits may recruit LTP-promoting proteins to the synapse (Barria & Malinow 2005).

The mechanism by which the NR2A/B ratio changes is determined by the length of dark exposure: short periods shift the ratio through increasing NR2B levels, whereas with longer periods NR2B protein levels return to normal and NR2A levels decrease (Chen & Bear 2007). To determine whether plasticity of the NR2A/B ratio is required for a shift in *θ*_m_, visual experience was manipulated in mice with a fixed ratio due to genetic deletion of NR2A (Philpot *et al*. 2007). The deletion of NR2A was found to both mimic and occlude the effects of dark rearing on the amplitude, decay kinetics and temporal summation of NMDA currents. Critically, when *θ*_m_ was examined by testing the frequency dependence of LTP and LTD, dark rearing failed to produce a shift in mice lacking NR2A, demonstrating a critical role for NR2A and the NR2A/B ratio in governing *θ*_m_ (Philpot *et al*. 2007).

If *θ*_m_ is modified via changing the NR2A/B ratio, and a change in *θ*_m_ is permissive for the potentiation of the open eye, the time course of the changes in the NR2A/B ratio (and therefore *θ*_m_) should slightly lead the time course of open-eye potentiation. When the NR2A/B ratio was examined in the mouse visual cortex following MD of the contralateral eye, a significant decrease in ratio was observed following 5 and 7 days of deprivation but not with shorter periods (Chen & Bear 2007). Given that open-eye potentiation during MD does not occur until after 5 days (Frenkel & Bear 2004), the observed time course is precisely as would be predicted.

## 5. LTP as a mechanism for open-eye potentiation

Although the strengthening of inputs originating from the open eye has been documented for over 30 years, the molecular mechanisms underlying this process have received scant attention relative to those mediating deprived-eye depression. Nonetheless, the predictions from the BCM theory are clear: open-eye inputs to the cortex, which remain at their original activity level during the early stages of MD, potentiate via homosynaptic mechanisms once *θ*_m_ drops below this activity level. LTP has been demonstrated at multiple cortical synapses *ex vivo*, and although the mechanisms appear to vary across layers similar to LTD (Wang & Daw 2003), homosynaptic NMDAR-dependent LTP has been shown at layer 3 synapses in the rat (Kirkwood & Bear 1994). Additionally, in rats, NMDAR-dependent LTP can be induced in layers 4 and 3 *in vivo* following tetanic stimulation of LGN, and this LTP is sufficient to increase the magnitude of visually evoked responses (Heynen & Bear 2001). These results suggest that homosynaptic LTP, possibly at thalamocortical synapses, can mimic the effects of open-eye potentiation after long-term MD.

Many manipulations known to disrupt homosynaptic LTP have been applied during OD plasticity (Daw *et al*. 2004; Hensch 2005; Hooks & Chen 2007), although many of these studies suffer from the inability of acute single-unit recordings to isolate changes in deprived-eye pathways from those serving the open eye. One example of this is the finding that OD plasticity is disrupted in mice with either disrupted αCaMKII autophosphorylation or lacking the protein entirely, which suggests a role for LTP (Gordon *et al*. 1996; Taha *et al*. 2002). Unfortunately, because all measures of OD in these studies were performed by comparing the relative drive from the deprived and non-deprived eyes, it is not clear which processes were disrupted.

Although the data on the mechanisms underlying open-eye potentiation in juvenile mice remain scarce, several related experiments are suggestive. Open-eye potentiation is absent in adult mice with a post-natal deletion of NR1 targeted to layers 2–4, suggesting that NMDAR-mediated plasticity plays a role (Sawtell *et al*. 2003). Further suggestion comes from the recently discovered phenomenon of stimulus-selective response potentiation (SRP). In juvenile mice, the magnitude of visually driven thalamocortical responses in layer 4 increases following repeated presentations of an oriented stimulus (Frenkel *et al*. 2006). This potentiation is specific to both the trained eye and the trained orientation, and is dependent on NMDAR activation. The discovery of SRP demonstrates that physiologically relevant potentiation of visual responses can occur *in vivo*, and shares a requirement for NMDAR activation with LTP.

If open-eye potentiation during MD occurs through LTP-like mechanisms, it is likely to be expressed through the delivery of AMPARs to synapses (Malinow *et al*. 2000), similar to the role we propose for AMPAR endocytosis and LTD in deprived-eye depression. It has been shown that the expression of a region of the GluR1 C-terminal tail is sufficient to both prevent the delivery of GluR1 to synapses and block LTP (Shi *et al*. 2001). Additionally, several studies in the amygdala, as well as the somatosensory and visual cortices, have shown that GluR1 delivery is required for experience-dependent plasticity occurring *in vivo* (Takahashi *et al*. 2003; Rumpel *et al*. 2005; Frenkel *et al*. 2006). If a similar blockade of GluR1 delivery could prevent potentiation of open-eye responses following 7 days of MD without affecting the decrease in deprived-eye responses, it would demonstrate that AMPAR insertion, and therefore probably LTP, is a necessary component subserving open-eye potentiation.

## 6. Alternative hypotheses for deprived-eye depression

Our view is that deprivation induces response depression via the mechanisms of LTD in layers 4 and 3, and that delayed response potentiation occurs via the mechanisms of LTP after permissive adjustment of the modification threshold. However, several alternative hypotheses have also been advanced to account for the phenomenology of OD plasticity.

Brief MD has been shown to lead to increased motility and a loss of dendritic spines located in superficial layers belonging to layer 5 pyramidal neurons in mice (Mataga *et al*. 2004; Oray *et al*. 2004). In both of these studies, the effect of MD on dendritic spine dynamics was found to be dependent on the tissue-type plasminogen activator (tPA)/plasmin proteolytic cascade. Brief MD elevates tPA activity in the cortex, and genetic deletion of tPA both reduces the magnitude of the OD shift assayed through single-unit recordings and prevents the loss of dendritic spines during MD (Mataga *et al*. 2002, 2004). Furthermore, spine motility can be increased with plasmin treatment, an effect that is occluded by prior MD (Oray *et al*. 2004). Together, these findings suggest that degradation of the extracellular matrix (ECM) by the tPA/plasmin cascade is an essential component of OD plasticity, possibly due to its role in promoting dendritic spine dynamics.

It is important to recognize, however, that these structural responses to MD are entirely consistent with the hypothesis that deprived-eye depression occurs through LTD mechanisms. It has been shown that LTD is associated with structural reorganization and a retraction of dendritic spines (Nagerl *et al*. 2004; Zhou *et al*. 2004; Bastrikova *et al*. 2008). Unfortunately, a significant limitation of current studies examining dendritic spines following MD is that it is unclear whether the observed changes are at spines receiving input from the open or deprived eye. Given that MD affects both deprived-eye and open-eye responses, it is critical to determine whether ECM degradation and increased spine motility relate to the depression or potentiation of visual responses.

A second recently proposed mechanism to account for the loss of deprived-eye responsiveness following MD during the critical period focuses on an increase in intracortical inhibition (Maffei *et al*. 2006). Using whole-cell recordings from connected pairs of neurons in the rat visual cortex, it was found that brief MD from P21 to P24 increased the inhibitory tone in the visual cortex by strengthening excitatory connections onto fast-spiking (FS) interneurons and also strengthening inhibitory connections from FS cells onto pyramidal neurons (Maffei *et al*. 2006). In the same study, it was found that this strengthening of inhibitory feedback could be achieved through a novel form of LTP of inhibition, which was occluded by prior MD. The increase in inhibitory drive following MD appears to be developmentally regulated, as deprivation in younger animals (P14–17) leads to decreased inhibition coupled with increased excitatory drive (Maffei *et al*. 2004).

**Figure 2:**
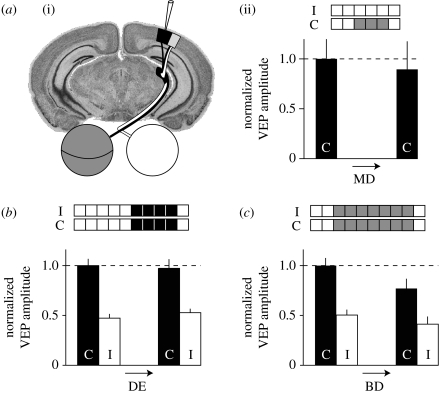
Layer 4 VEPs display binocular competition but not synaptic scaling. (*a*) MD fails to elicit changes in responses recorded in (i) the monocular zone ((ii) record C VEPs), and (*b*) 4 days of complete darkness fail to modify responses in the binocular zone, showing that deprived-eye depression is impaired in neurons that do not experience binocular competition (record C and I VEPs). (*c*) Prolonged binocular deprivation fails to produce scaling up of VEPs recorded in layer 4 of the binocular cortex. Responses to stimulation of the contralateral (C) and ipsilateral (I) eyes were recorded prior to and following 7 days of binocular lid suture. No change in the response to either eye was observed, contrary to the prediction that synaptic scaling would lead to increased responses following decreased input activity. Experimental treatment: white, no manipulation; grey, eyelid suture; black, dark exposure. Data are replotted from (*a*) Frenkel & Bear (2008), (*b*) Blais *et al*. (2008) and (*c*) Frenkel & Bear (2004).

The relevance of these experiments to OD plasticity in mice is unclear as they were performed in the monocular zone of rat visual cortex, which lacks input from the open eye, and therefore binocular interactions. In the mouse, a period of MD that is sufficient to cause maximal deprived-eye depression in the binocular zone (3 days) has no effect on VEPs in the monocular segment (Frenkel & Bear 2008). Similarly, 4 days of complete darkness has no effect on VEP amplitude in the binocular segments (Blais *et al*. 2008; figure 2*a*,*b*).

VEPs may reflect the strength of feed-forward geniculocortical transmission, and therefore be insensitive to intracortical modifications. However, it is still unclear how a rise in inhibitory tone could account for the specific weakening of deprived-eye responses in the binocular zone during MD. Given the lack of OD columns in rats, lateral inhibition of neighbouring columns cannot occur; therefore, such a model requires the existence of eye-specific inhibitory networks within layer 4 of the binocular zone. The existence of such networks is unlikely based on the mixing of eye-specific afferents in the binocular visual cortex of rodents—there are few, if any, neurons that receive input exclusively from the ipsilateral eye.

## 7. Alternative hypotheses for open-eye potentiation

Homeostatic mechanisms have long been thought to play a role in the response to altered sensory experience. In fact, the BCM sliding threshold model describes a means to achieve the homeostasis of firing rates in the face of decreased synaptic drive as the modification threshold will adopt whatever position is required to maintain the firing rate by adjusting synaptic weights via LTP or LTD. In this model, prolonged MD leads to a decrease in *θ*_m_, which facilitates LTP of open-eye inputs, thereby increasing synaptic drive and restoring postsynaptic firing rates closer to their original position.

An alternative mechanism of homeostatic regulation is synaptic scaling, which was first described in dissociated rat cortical cultures, where blockade of activity with TTX results in the global multiplicative scaling up of synaptic weights (Turrigiano *et al*. 1998). It has been proposed that such a mechanism may account for the strengthening of open-eye responses following MD (Turrigiano & Nelson 2004). Visual deprivation, either through monocular inactivation via intraocular TTX or dark rearing, increases mEPSC amplitudes recorded in layer 2/3 neurons in juvenile rats (Desai *et al*. 2002; Goel *et al*. 2006).

Recent work using *in vivo* calcium imaging to measure visual responses in mice has suggested that similar mechanisms are also invoked during MD. In addition to the expected shift in responses towards open-eye dominance, responses of cells driven exclusively by the deprived eye were larger following MD (Mrsic-Flogel *et al*. 2007). However, the results of this study share a similar limitation with acute single-unit recordings, namely that it is impossible to measure responses in the same cells before and after a manipulation, necessitating between-group comparisons and relative measures of responsiveness. Therefore, it is not clear whether previously monocular cells driven by the deprived eye undergo response potentiation, or originally binocular cells become increasingly monocular.

**Figure 3:**
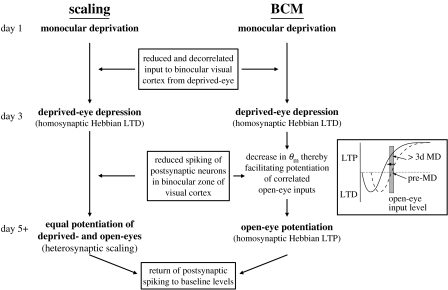
Alternative mechanisms for achieving open-eye potentiation and homeostasis following MD. Under both the synaptic scaling and BCM hypotheses, the initial response to MD is the depression of deprived-eye responses, which results from the decorrelation of deprived-eye inputs following lid suture. This decorrelation of input activity drives homosynaptic LTD in the binocular visual cortex. A consequence of the degraded input coupled with the weakening of synapses via LTD is the reduced spiking activity of neurons in the binocular visual cortex, which, after several days, leads to compensatory changes. The nature of these changes differs between the two models of open-eye potentiation. According to the synaptic scaling model, neurons respond to reduced spiking activity by globally scaling up synaptic weights, thereby increasing incoming drive and returning spiking to baseline levels. By contrast, under the BCM model, neurons respond to decreased spiking activity by lowering the modification threshold (*θ*_m_), thus promoting LTP across a larger range of inputs. The inset shows that prior to MD, the boundary between potentiating and depressing inputs is equal to the open-eye input level, thereby maintaining stable open-eye responses if visual experience is not manipulated. Following 3 days of MD, the boundary shifts to the left, and open-eye inputs to the cortex (the strength of which has not changed as visual experience through the open eye has remained constant) now induce homosynaptic potentiation. This increase in drive from the open eye in turn elevates postsynaptic spiking to baseline levels. Therefore, both the synaptic scaling and BCM models can account for both the potentiation of open-eye responses and the output homeostasis of visual cortical neurons. The difference lies in the behaviour of the deprived-eye inputs during the later stages of MD. Synaptic scaling is heterosynaptic; therefore, both open- and deprived-eye inputs are predicted to increase equally. By clear contrast, BCM-mediated homeostasis occurs via homosynaptic mechanisms, thus only open-eye responses potentiate. Current data suggest that the deprived eye does not potentiate proportionally to the open eye, suggesting that global scaling of responses is not occurring in response to MD.

Given that both the BCM and synaptic scaling models describe homeostatic mechanisms for maintaining a given level of postsynaptic activity, we should move beyond the artificial distinction often seen in the literature between ‘Hebbian’ and ‘homeostatic’ plasticity mechanisms in the context of OD plasticity. A more useful distinction, which is supported by both theoretical and experimental work, is between homeostatic mechanisms expressed globally (synaptic scaling) as opposed to homosynaptically (BCM; figure 3).

One possible way to distinguish between homosynaptic BCM and heterosynaptic scaling models is in the predicted response to binocular deprivation (BD). A synaptic scaling model predicts that the reduction in visual drive from both eyes should lead to the potentiation of responses following BD, whereas no change in responsiveness is predicted by BCM-based models (Blais *et al*. 1999; Blais *et al*. 2008). Unfortunately, the available data are contradictory. Visual responses measured with *in vivo* calcium imaging in layer 2/3 were potentiated following BD (Mrsic-Flogel *et al*. 2007), although a similar increase was not observed in previous studies using either single-unit recordings across cortical layers (Gordon & Stryker 1996) or chronic VEP recordings in layer 4 (Frenkel & Bear 2004; Blais *et al*. 2008; figure 2*c*).

The NMDAR dependence of open-eye potentiation may provide a second means to distinguish between synaptic scaling and LTP. Homosynaptic LTP at layer 4 to 2/3 synapses as well as at layer 2/3 to 5 synapses requires NMDAR activation (Kirkwood *et al*. 1993; Wang & Daw 2003), whereas synaptic scaling in culture does not (Turrigiano *et al*. 1998; Turrigiano & Nelson 2004). Open-eye potentiation is absent in mice lacking NR1 in layers 2–4 (Sawtell *et al*. 2003), suggesting that homosynaptic LTP may underlie the strengthening of open-eye responses.

An additional method to distinguish these two models is through the use of genetically modified animals deficient in synaptic scaling. This approach has recently been used by Kaneko *et al*. (2008) who studied OD plasticity in mice lacking tumour necrosis factor-α (TNFα), which fail to show scaling up of synaptic responses following decreased activity *in vitro*. Using repeated imaging of intrinsic optical signals, it was found that open-eye potentiation similarly fails to occur in the absence of TNFα. The finding of normal LTP in layer 2/3 of these mice strongly suggests that synaptic scaling may drive open-eye potentiation. One problem with this interpretation, as has been pointed out by Aizenman & Pratt (2008), is that deprived-eye responses did not increase proportionally to open-eye responses during the later stages of MD (Kaneko *et al*. 2008). Likewise, at the behavioural level, delayed increases in open-eye acuity are not accompanied by parallel increases in deprived-eye acuity (Iny *et al*. 2006). A key feature of synaptic scaling is that *all* synapses are scaled up or down equally, a feature that is essential to prevent information loss and preserve the relative strengths of distinct inputs (Turrigiano *et al*. 1998). Therefore, the disproportionate effect on open-eye responses during the later stages of MD argues against synaptic scaling as the sole mechanism of open-eye potentiation.

At this time, it seems reasonable to suggest that the discordant findings from imaging versus electrophysiology and behaviour may arise from significant laminar differences in the cortical response to MD and BD. Layer 4 neurons receiving convergent thalamocortical inputs that are dedicated to each eye might maintain homeostasis via a homosynaptic BCM-type rule, in which a loss of strength of one input is compensated for by an increase in strength by a competing input. Conversely, neurons in the superficial layers that do not receive segregated inputs from the two eyes might maintain responsiveness in the face of deprived-eye depression via a heterosynaptic scaling mechanism.

## 8. Outstanding issues in OD plasticity

A clear challenge still facing the field of OD plasticity is to demonstrate a causal role for specific molecular processes in response to MD. Several mechanisms have emerged as potential contributors to deprived-eye depression, but it remains to be determined which, if any, are required. Similarly, evidence suggests that a decrease in the NR2A/B ratio may facilitate open-eye potentiation, but again a causal role has yet to be demonstrated. Viral-mediated overexpression of NR2A or RNAi knockdown of NR2B in the visual cortex specifically during the period of MD may be able to test this by preventing a decrease in the NR2A/B ratio.

LTP provides a tempting mechanistic framework for open-eye potentiation, but many questions remain. Local disruption of either NMDAR function or synaptic AMPAR insertion specifically during the later stages of MD may help distinguish processes serving deprived-eye depression from those involved in open-eye potentiation. Additionally, it is important to determine whether open-eye potentiation occludes subsequent LTP, and to probe the relative contributions of homo- and heterosynaptic processes to open-eye potentiation.

It is essential that future studies of OD plasticity recognize the numerous differences in synaptic plasticity across cortical layers. This is especially important when manipulating molecular pathways during MD, as those pathways may be involved only in OD plasticity in specific layers. Greater attention must be given to the laminar position of neurons recorded with single-unit techniques, as grouping neurons across layers may obscure effects of manipulations that are layer specific.

Experimental techniques for characterizing OD plasticity have advanced greatly over the last four decades. For example, the longitudinal within-animal observations of visual responses afforded by chronic VEP recordings have greatly added to the understanding of the kinetics of OD plasticity. Currently, all available experimental techniques have significant limitations: single-unit recordings and calcium imaging offer single-cell resolution, but do not allow for chronic recordings, whereas VEP recordings allow for chronic measurements, but lack single-cell resolution. Recent advances in transgenic calcium sensors (reviewed in Knopfel *et al*. 2006) may provide a solution, allowing large numbers of neurons to be observed repeatedly over the course of MD. The combination of transgenic calcium sensors with virally mediated disruption of specific molecular pathways should allow the investigation of whether individual neurons in a cortical network respond to MD in a cell-autonomous manner.

An additional long-standing challenge in the field has been to clearly label eye-specific inputs into the cortex in living tissue. Without this information it is difficult to interpret many of the results in the literature. For example, MD produces changes in dendritic spines (Mataga *et al*. 2004; Oray *et al*. 2004), but it is unclear whether these changes are restricted to spines receiving input from a particular eye. Likewise, it remains to be determined whether the occlusion of LTD by prior MD (Crozier *et al*. 2007) is restricted to deprived-eye but not open-eye inputs into layer 4, as would be predicted.

In this review, we have focused on the mechanisms underlying the physiological response to MD, and have not yet addressed the developmental regulation of OD plasticity. Classically, OD plasticity has been described as developmentally restricted to a critical period in early post-natal life, and evidence still supports this view in many species, including rats, cats and monkeys. In mice, on the other hand, OD plasticity has been demonstrated throughout adulthood (Tagawa *et al*. 2005; Frenkel *et al*. 2006; Hofer *et al*. 2006), indicating that the critical period concept is not applicable to all species. Although the response to MD occurs more slowly in adult mice, this plasticity appears qualitatively indistinguishable from that in juvenile animals (Frenkel *et al*. 2006).

However, the potential for adult OD plasticity is definitely not restricted to mice. For example, recent work has shown that under certain conditions, rats, which have a critical period, can exhibit rapid and robust OD plasticity as adults. Degradation of chondroitin sulphate proteoglycans in the ECM can restore a rapid OD shift in adult rats (Pizzorusso *et al*. 2002), as can oral administration of the antidepressant fluoxetine (Maya Vetencourt *et al*. 2008). Even subtle manipulations of experience are sufficient: both brief dark exposure (He *et al*. 2006, 2007) and environmental enrichment (He *et al*. 2007; Sale *et al*. 2007) are able to restore OD plasticity in adult rats. These newer findings build on seminal work performed in cats almost 30 years ago showing that OD plasticity can be restored by local infusion of noradrenaline into adult visual cortex (Kasamatsu *et al*. 1979). The restoration of plasticity in adult animals is especially significant from a therapeutic perspective, as dark rearing, environmental enrichment and fluoxetine all promote the recovery of vision following chronic deprivation amblyopia.

The findings of OD plasticity in adult animals raise two important questions. First, are the mechanisms qualitatively different from those active in juveniles? Second, is there a final common pathway (e.g. altered inhibition or NMDAR subunit composition) affected by the manipulations that promote plasticity in adult animals? Answering these two questions will represent a major advance in the treatment of amblyopia, and may provide insights into other forms of developmentally regulated plasticity, including learning and memory.

## 9. Conclusion

Advances in recording techniques, coupled with the development of molecular tools and transgenic mice, have led to the identification of many of the molecular pathways involved in OD plasticity. Based on currently available evidence, we favour a three-phase model of OD plasticity in layer 4 of mice: deprived-eye responses rapidly weaken following MD through homosynaptic LTD, while over a longer time period the threshold for synaptic modification is lowered, facilitating the strengthening of open-eye responses via homosynaptic LTP. In addition to presenting several directly testable hypotheses concerning OD plasticity in mice, this model suggests potential therapeutic strategies for amblyopia in humans.

## References

[bib1] Abraham W.C., Bear M.F. (1996). Metaplasticity: the plasticity of synaptic plasticity. Trends Neurosci.

[bib2] Aizenman C.D., Pratt K.G. (2008). There's more than one way to scale a synapse. Neuron.

[bib3] Barria A., Malinow R. (2005). NMDA receptor subunit composition controls synaptic plasticity by regulating binding to CaMKII. Neuron.

[bib4] Bastrikova N., Gardner G.A., Reece J.M., Jeromin A., Dudek S.M. (2008). Synapse elimination accompanies functional plasticity in hippocampal neurons. Proc. Natl Acad. Sci. USA.

[bib5] Bear M.F. (2003). Bidirectional synaptic plasticity: from theory to reality. Phil. Trans. R. Soc. B.

[bib6] Bear M.F., Cooper L.N., Ebner F.F. (1987). A physiological basis for a theory of synapse modification. Science.

[bib7] Bienenstock E.L., Cooper L.N., Munro P.W. (1982). Theory for the development of neuron selectivity: orientation specificity and binocular interaction in visual cortex. J. Neurosci.

[bib8] Blais B., Frenkel M., Kuindersma S., Muhammad R., Shouval H.Z., Cooper L.N., Bear M.F. (2008). Recovery from monocular deprivation using binocular deprivation. J. Neurophysiol.

[bib9] Blais B.S., Shouval H.Z., Cooper L.N. (1999). The role of presynaptic activity in monocular deprivation: comparison of homosynaptic and heterosynaptic mechanisms. Proc. Natl Acad. Sci. USA.

[bib10] Carmignoto G., Vicini S. (1992). Activity-dependent decrease in NMDA receptor responses during development of the visual cortex. Science.

[bib11] Chen W.S., Bear M.F. (2007). Activity-dependent regulation of NR2B translation contributes to metaplasticity in mouse visual cortex. Neuropharmacology.

[bib12] Choi S.Y., Morales B., Lee H.K., Kirkwood A. (2002). Absence of long-term depression in the visual cortex of glutamic Acid decarboxylase-65 knock-out mice. J. Neurosci.

[bib13] Crozier R.A., Wang Y., Liu C.H., Bear M.F. (2007). Deprivation-induced synaptic depression by distinct mechanisms in different layers of mouse visual cortex. Proc. Natl Acad. Sci. USA.

[bib14] Daw N., Rao Y., Wang X.F., Fischer Q., Yang Y. (2004). LTP and LTD vary with layer in rodent visual cortex. Vision Res.

[bib15] Desai N.S., Cudmore R.H., Nelson S.B., Turrigiano G.G. (2002). Critical periods for experience-dependent synaptic scaling in visual cortex. Nat. Neurosci.

[bib16] Deshmukh S., Onozuka K., Bender K.J., Bender V.A., Lutz B., Mackie K., Feldman D.E. (2007). Postnatal development of cannabinoid receptor type 1 expression in rodent somatosensory cortex. Neuroscience.

[bib17] Doshi N.R., Rodriguez M.L. (2007). Amblyopia. Am. Fam. Physician.

[bib18] Fischer Q.S., Beaver C.J., Yang Y., Rao Y., Jakobsdottir K.B., Storm D.R., Mcknight G.S., Daw N.W. (2004). Requirement for the RIIbeta isoform of PKA, but not calcium-stimulated adenylyl cyclase, in visual cortical plasticity. J. Neurosci.

[bib19] Flint A.C., Maisch U.S., Weishaupt J.H., Kriegstein A.R., Monyer H. (1997). NR2A subunit expression shortens NMDA receptor synaptic currents in developing neocortex. J. Neurosci.

[bib20] Frenkel M.Y., Bear M.F. (2004). How monocular deprivation shifts ocular dominance in visual cortex of young mice. Neuron.

[bib21] Frenkel M.Y., Bear M.F., Chalupa L.M., Williams R.W. (2008). Bidirectionlal experience-dependent plasticity in primary visual cortex. Eye, retina, and visual system of the mouse.

[bib22] Frenkel M.Y., Sawtell N.B., Diogo A.C., Yoon B., Neve R.L., Bear M.F. (2006). Instructive effect of visual experience in mouse visual cortex. Neuron.

[bib23] Goel A., Jiang B., Xu L.W., Song L., Kirkwood A., Lee H.K. (2006). Cross-modal regulation of synaptic AMPA receptors in primary sensory cortices by visual experience. Nat. Neurosci.

[bib25] Gordon J.A., Stryker M.P. (1996). Experience-dependent plasticity of binocular responses in the primary visual cortex of the mouse. J. Neurosci.

[bib24] Gordon J.A., Cioffi D., Silva A.J., Stryker M.P. (1996). Deficient plasticity in the primary visual cortex of alpha-calcium/calmodulin-dependent protein kinase II mutant mice. Neuron.

[bib26] He H.Y., Hodos W., Quinlan E.M. (2006). Visual deprivation reactivates rapid ocular dominance plasticity in adult visual cortex. J. Neurosci.

[bib27] He H.Y., Ray B., Dennis K., Quinlan E.M. (2007). Experience-dependent recovery of vision following chronic deprivation amblyopia. Nat. Neurosci.

[bib28] Hensch T.K. (2005). Critical period plasticity in local cortical circuits. Nat. Rev. Neurosci.

[bib29] Hensch T.K., Fagiolini M., Mataga N., Stryker M.P., Baekkeskov S., Kash S.F. (1998a). Local GABA circuit control of experience-dependent plasticity in developing visual cortex. Science.

[bib30] Hensch T.K., Gordon J.A., Brandon E.P., Mcknight G.S., Idzerda R.L., Stryker M.P. (1998b). Comparison of plasticity *in vivo* and *in vitro* in the developing visual cortex of normal and protein kinase A RIbeta-deficient mice. J. Neurosci.

[bib31] Heynen A.J., Bear M.F. (2001). Long-term potentiation of thalamocortical transmission in the adult visual cortex *in vivo*. J. Neurosci.

[bib32] Heynen A.J., Yoon B.J., Liu C.H., Chung H.J., Huganir R.L., Bear M.F. (2003). Molecular mechanism for loss of visual cortical responsiveness following brief monocular deprivation. Nat. Neurosci.

[bib33] Hofer S.B., Mrsic-Flogel T.D., Bonhoeffer T., Hubener M. (2006). Lifelong learning: ocular dominance plasticity in mouse visual cortex. Curr. Opin. Neurobiol.

[bib34] Hooks B.M., Chen C. (2007). Critical periods in the visual system: changing views for a model of experience-dependent plasticity. Neuron.

[bib35] Iny K., Heynen A.J., Sklar E., Bear M.F. (2006). Bidirectional modifications of visual acuity induced by monocular deprivation in juvenile and adult rats. J. Neurosci.

[bib36] Kaneko M., Stellwagen D., Malenka R.C., Stryker M.P. (2008). Tumor necrosis factor-alpha mediates one component of competitive, experience-dependent plasticity in developing visual cortex. Neuron.

[bib37] Kasamatsu T., Pettigrew J.D., Ary M. (1979). Restoration of visual cortical plasticity by local microperfusion of norepinephrine. J. Comp. Neurol.

[bib38] Kirkwood A., Bear M.F. (1994). Hebbian synapses in visual cortex. J. Neurosci.

[bib39] Kirkwood A., Dudek S.M., Gold J.T., Aizenman C.D., Bear M.F. (1993). Common forms of synaptic plasticity in the hippocampus and neocortex *in vitro*. Science.

[bib40] Kirkwood A., Rioult M.C., Bear M.F. (1996). Experience-dependent modification of synaptic plasticity in visual cortex. Nature.

[bib41] Knopfel T., Diez-Garcia J., Akemann W. (2006). Optical probing of neuronal circuit dynamics: genetically encoded versus classical fluorescent sensors. Trends Neurosci.

[bib42] Liu C.H., Heynen A.J., Shuler M.G., Bear M.F. (2008). Cannabinoid receptor blockade reveals parallel plasticity mechanisms in different layers of mouse visual cortex. Neuron.

[bib43] Maffei A., Nataraj K., Nelson S.B., Turrigiano G.G. (2006). Potentiation of cortical inhibition by visual deprivation. Nature.

[bib44] Maffei A., Nelson S.B., Turrigiano G.G. (2004). Selective reconfiguration of layer 4 visual cortical circuitry by visual deprivation. Nat. Neurosci.

[bib45] Malenka R.C., Bear M.F. (2004). LTP and LTD: an embarrassment of riches. Neuron.

[bib46] Malinow R., Mainen Z.F., Hayashi Y. (2000). LTP mechanisms: from silence to four-lane traffic. Curr. Opin. Neurobiol.

[bib48] Mataga N., Nagai N., Hensch T.K. (2002). Permissive proteolytic activity for visual cortical plasticity. Proc. Natl Acad. Sci. USA.

[bib47] Mataga N., Mizuguchi Y., Hensch T.K. (2004). Experience-dependent pruning of dendritic spines in visual cortex by tissue plasminogen activator. Neuron.

[bib49] Maya Vetencourt J.F., Sale A., Viegi A., Baroncelli L., De Pasquale R., O'leary O.F., Castren E., Maffei L. (2008). The antidepressant fluoxetine restores plasticity in the adult visual cortex. Science.

[bib50] Mioche L., Singer W. (1989). Chronic recordings from single sites of kitten striate cortex during experience-dependent modifications of receptive-field properties. J. Neurophysiol.

[bib51] Monyer H., Burnashev N., Laurie D.J., Sakmann B., Seeburg P.H. (1994). Developmental and regional expression in the rat brain and functional properties of four NMDA receptors. Neuron.

[bib52] Mrsic-Flogel T.D., Hofer S.B., Ohki K., Reid R.C., Bonhoeffer T., Hubener M. (2007). Homeostatic regulation of eye-specific responses in visual cortex during ocular dominance plasticity. Neuron.

[bib53] Nagerl U.V., Eberhorn N., Cambridge S.B., Bonhoeffer T. (2004). Bidirectional activity-dependent morphological plasticity in hippocampal neurons. Neuron.

[bib54] Oray S., Majewska A., Sur M. (2004). Dendritic spine dynamics are regulated by monocular deprivation and extracellular matrix degradation. Neuron.

[bib57] Philpot B.D., Sekhar A.K., Shouval H.Z., Bear M.F. (2001). Visual experience and deprivation bidirectionally modify the composition and function of NMDA receptors in visual cortex. Neuron.

[bib56] Philpot B.D., Espinosa J.S., Bear M.F. (2003). Evidence for altered NMDA receptor function as a basis for metaplasticity in visual cortex. J. Neurosci.

[bib55] Philpot B.D., Cho K.K., Bear M.F. (2007). Obligatory role of NR2A for metaplasticity in visual cortex. Neuron.

[bib58] Pizzorusso T., Medini P., Berardi N., Chierzi S., Fawcett J.W., Maffei L. (2002). Reactivation of ocular dominance plasticity in the adult visual cortex. Science.

[bib59] Prusky G.T., West P.W., Douglas R.M. (2000). Experience-dependent plasticity of visual acuity in rats. Eur. J. Neurosci.

[bib60] Quinlan E.M., Olstein D.H., Bear M.F. (1999a). Bidirectional, experience-dependent regulation of *N*-methyl-d-aspartate receptor subunit composition in the rat visual cortex during postnatal development. Proc. Natl Acad. Sci. USA.

[bib61] Quinlan E.M., Philpot B.D., Huganir R.L., Bear M.F. (1999b). Rapid, experience-dependent expression of synaptic NMDA receptors in visual cortex *in vivo*. Nat. Neurosci.

[bib62] Rao Y., Daw N.W. (2004). Layer variations of long-term depression in rat visual cortex. J. Neurophysiol.

[bib63] Rao Y., Fischer Q.S., Yang Y., Mcknight G.S., Larue A., Daw N.W. (2004). Reduced ocular dominance plasticity and long-term potentiation in the developing visual cortex of protein kinase A RII alpha mutant mice. Eur. J. Neurosci.

[bib64] Rittenhouse C.D., Siegler B.A., Voelker C.C., Shouval H.Z., Paradiso M.A., Bear M.F. (2006). Stimulus for rapid ocular dominance plasticity in visual cortex. J. Neurophysiol.

[bib65] Rumpel S., Ledoux J., Zador A., Malinow R. (2005). Postsynaptic receptor trafficking underlying a form of associative learning. Science.

[bib66] Sale A., Maya Vetencourt J.F., Medini P., Cenni M.C., Baroncelli L., De Pasquale R., Maffei L. (2007). Environmental enrichment in adulthood promotes amblyopia recovery through a reduction of intracortical inhibition. Nat. Neurosci.

[bib67] Sawtell N.B., Frenkel M.Y., Philpot B.D., Nakazawa K., Tonegawa S., Bear M.F. (2003). NMDA receptor-dependent ocular dominance plasticity in adult visual cortex. Neuron.

[bib68] Shi S., Hayashi Y., Esteban J.A., Malinow R. (2001). Subunit-specific rules governing AMPA receptor trafficking to synapses in hippocampal pyramidal neurons. Cell.

[bib69] Tagawa Y., Kanold P.O., Majdan M., Shatz C.J. (2005). Multiple periods of functional ocular dominance plasticity in mouse visual cortex. Nat. Neurosci.

[bib70] Taha S., Hanover J.L., Silva A.J., Stryker M.P. (2002). Autophosphorylation of alphaCaMKII is required for ocular dominance plasticity. Neuron.

[bib71] Takahashi T., Svoboda K., Malinow R. (2003). Experience strengthening transmission by driving AMPA receptors into synapses. Science.

[bib73] Turrigiano G.G., Nelson S.B. (2004). Homeostatic plasticity in the developing nervous system. Nat. Rev. Neurosci.

[bib72] Turrigiano G.G., Leslie K.R., Desai N.S., Rutherford L.C., Nelson S.B. (1998). Activity-dependent scaling of quantal amplitude in neocortical neurons. Nature.

[bib74] Wang X.F., Daw N.W. (2003). Long term potentiation varies with layer in rat visual cortex. Brain Res.

[bib75] Zhou Q., Homma K.J., Poo M.M. (2004). Shrinkage of dendritic spines associated with long-term depression of hippocampal synapses. Neuron.

